# Enhanced Bioremediation of Aged Polycyclic Aromatic Hydrocarbons in Soil Using Immobilized Microbial Consortia Combined with Strengthening Remediation Strategies

**DOI:** 10.3390/ijerph20031766

**Published:** 2023-01-18

**Authors:** Haixuan Zhou, Xiurong Gao, Suhang Wang, Youchi Zhang, Frederic Coulon, Chao Cai

**Affiliations:** 1 Key Lab of Urban Environment and Health, Institute of Urban Environment, Chinese Academy of Sciences, Xiamen 361021, China; 2 University of Chinese Academy of Sciences, Beijing 100049, China; 3 School of Water, Energy and Environment, Cranfield University, Cranfield MK43 0AL, UK

**Keywords:** PAH degradation, bacterial consortia, immobilization, strengthening remediation

## Abstract

Microbial biodegradation is considered as one of the most effective strategies for the remediation of soil contaminated with polycyclic aromatic hydrocarbons (PAHs). To improve the degradation efficiency of PAHs, PAH-degrading consortia combined with strengthening remediation strategies was used in this study. The PAH biodegrading performance of seven bacterial consortia constructed by different ratios of *Mycobacterium gilvum* MI, *Mycobacterium* sp. ZL7 and *Rhodococcus rhodochrous* Q3 was evaluated in an aqueous system containing phenanthrene, pyrene, benzo[a]pyrene and benzo[b]fluoranthene. Bacterial consortium H6 (Q3:ZL7:MI = 1:2:2) performed a high degrading efficiency of 59% in 8 days. The H6 was subsequently screened to explore its potential ability and performance to degrade aged PAHs in soils from a coking plant and the effects of strengthening strategies on the aged PAH degradation, including the addition of glucose or sodium dodecyl benzene sulfonate (SDBS) individually or as a mixture along immobilization of the inoculant on biochar. The highest degradation efficiencies, which were 15% and 60% for low-molecular-weight (LMW) PAHs and high-molecular-weight (HMW) PAHs, respectively, were observed in the treatment using immobilized microbial consortium H6 combined with the addition of glucose and SDBS after 24 days incubation. This study provides new insights and guidance for future remediation of aged PAH contaminated soils.

## 1. Introduction

Polycyclic aromatic hydrocarbons (PAHs) are ubiquitous hydrophobic organic pollutants in the environment, with genotoxicity, carcinogenicity, mutagenicity, and teratogenicity. PAHs have long-term health effects even at low levels, such as cataracts, kidney and liver damage, and jaundice, which threaten both human and ecosystem health [1,2]. The aqueous solubility of PAHs decreases with the increasing number of benzene rings [3]. After entering the into soil, PAHs can sorb on and become entrapped within mineral or organic matter particles with increasing contact time and then PAHs become unavailable and aging. PAHs are less susceptible to biodegradation especially for the high-molecular-weight (HMW) PAHs due to their rapidly aging state with decreasing bioavailability [4]. Furthermore, PAH molecules have greater resonance stabilization and become inherently more difficult to degrade with the increasing number of benzene rings [5]. Many previous works have developed effective techniques for remediating aged PAHs in soils under laboratory simulation conditions. However, the remediation effects of these techniques in real aged PAHs contaminated soils are not fully understood.

Bioremediation has been regarded as an efficient and sustainable approach for remediation of PAHs in soils due to the advantages of low energy consumption, low secondary pollution, and cost-effectiveness [6,7]. However, it is difficult for microorganisms to attack aged PAHs due to their low bioavailability. To overcome this problem, strategies to optimize microbial capacity to perform biodegradation have been explored by considering both single strain and mixed consortia [8,9]. Guo et al. isolated a pyrene (PYR) degrading bacterial consortia from contaminated soil using PYR as the sole carbon source [10]. The isolates degraded 76% of PYR in a liquid matrix within 10 days. Xiong et al. isolated *Mycobacterium gilvum* strain from PAHs contaminated soil and found it was able to remove 98% of PYR in 5 days [11]. Despite the ability to degrade a specific PAH compound, notably, hydrocarbon contamination is often composed of several hundreds of compounds, which increases the difficulty of microbial degradation [9]. It has been reported that single species inoculant has limited substrate ranges and can only degrade one or two kinds of PAHs [12]. In contrast, multi-species bacterial consortia have been reported to provide symbiotic proliferation and synergistic metabolism and can form an adapted and stable micro-ecosystem to degrade a wide range of organic pollutants [13,14,15]. Several studies have assessed the efficiency of microbial consortia to remediate PAHs contaminated soils. For example, Wanapaisan et al. prepared a PYR degrading microbial consortium containing five bacteria that enhanced PYR biodegradation due to synergistic activities of the bacterial consortia [16]. Similarly, Zafra et al. constructed a bacterial consortium that can remove phenanthrene (PHE), PYR, and benzo[a]pyrene (BaP), each present in soil at 333 mg/kg, by 92%, 64% and 65%, respectively, within 14 days [17]. More in-depth research should be carried out on the remediation of PAHs contaminated soil by microbial consortia, especially for the remediation effect in real soil.

Many strengthening strategies have been applied to improve the degradation efficiency of PAHs by microorganisms. The addition of surfactant has been shown to be efficient for enhancing the solubility of PAHs and thereby improving their rate of biodegradation [18,19,20,21]. The addition of carbon sources and/or nutrients has also been reported as an effective approach to enhance bioremediation of PAHs [22,23]. Immobilized microorganism technology is an effective tool for improving the efficiency of microbial remediation, for example, retaining microorganisms in the soil for a long time, improving the activity of microorganisms, and decreasing the sensitivity of microorganisms to environmental conditions [24,25]. Previous studies have shown that immobilization of PAH degrading bacteria onto high-affinity sorbents for PAHs can improve PAH remediation performance [26,27]. Biochar is often used as sorbent amendments due to its excellent properties, such as high surface area and porosity (making it a good candidate for microbial habitats), low-cost and an opportunity to resourcefully use organic waste [28,29]. However, the comprehensive effects of various strengthening remediation strategies still need to be further explored.

To date, there are few reports about the combination of different strengthening remediation strategies, i.e., microbial consortia, immobilization with strong sorbent, and the use of surfactants and labile carbon sources to enhance biodegradation efficiency of aged PAHs in soil. In the present study, seven PAH degrading bacterial consortia containing *Rhodococcus* and *Mycobacterium* genera were constructed and evaluated in liquid media and contaminated soils. The goals of this study were to (1) evaluate the bioremediation efficiency of real aged PAH contaminated soil collected from a coke plant using efficient microbial consortium; (2) investigate the effect of the combination of strengthening strategies on the bioremediation efficiency of PAHs. This study constructed highly efficient artificial microbial consortium and explored the remediation effects of different strengthening strategies, which provide technical support for bioremediation of aging PAHs contaminated soil.

## 2. Materials and Methods

### 2.1. Soil Samples and PAHs Degrading Strains

Soil samples were collected from a former coking plant (CP) (N 39°51′0.42″, E 116°31′38.83″) in Beijing, China. The soil had been contaminated with PAHs for more than 50 years and the PAHs had therefore undergone a long aging process. The average total concentration of 16 US EPA PAHs is 255 ± 22 mg/kg (see Supplementary Materials Table S1 for quantification details). The concentration of the LMW PAHs, including naphthalene, acenaphthylene, acenaphthene, fluorene, phenanthrene, anthracene, fluoranthene and pyrene, is 62 ± 10 mg/kg. The concentration of HMW PAHs, including benzo[a]anthracene, chrysene, benzo[b]fluoranthene (BbF), benzo[k]fluoranthene, benzo[a]pyrene, indeno [1,2,3-c,d] pyrene, dibenzo[a,h]anthracene and benzo[g,h,i]perylene, is 193 ± 13 mg/kg. The proportion of HMW PAH is higher than 75%. Soil physical and chemical properties are described in Supplementary Materials for method details.

*Mycobacterium gilvum* (MI), *Rhodococcus rhodochrous* (Q3) and *Mycobacterium* sp. (ZL7) were previously isolated from soil as described by Ping et al. [30]. Strains were identified by 16S rRNA gene sequencing with primer 27F (5′-AGAGTTTGATCCTGGCTCAG-3′) and 1492R (5′-TACGGCTACCTTGTTACGACTT-3′), with identity similarity values of at least 99% in NCBI. At present, the patents for these strains have been applied, and China General Microbiological Culture Collection Center preserved them with the identifiers 16,445, 16,446, and 10,941. PYR was used as the sole carbon and energy source for screening the three strains. The strains were separated and purified on solid LB medium. Antagonistic effects among the three strains were tested on solid LB medium, according to the method described by Fuentes et al. [31]. The antagonistic effect between two strains was demonstrated by growth inhibition, which occurred at the intersection of two strains, and all pairwise comparisons were considered.

### 2.2. Construction and Optimization of PAHs Degrading Bacterial Consortia 

The bacterial suspension was prepared as follows: (1) the strains were inoculated in liquid LB medium and cultured at 37 °C, 180 rpm to logarithmic phase (OD_600_ = 1.0, the length of light path is 1 cm). (2) Then, the bacterial culture was centrifuged at 3500 rpm for 10 min; the supernatant was discarded and sterilized minimal salt medium (MSM) was added to resuspend the strains. (3) The suspension was adjusted to the required cell density (OD_600_ = 1.0) using MSM. Then, the suspensions of Q3, ZL7 and MI were mixed in different volume ratios to establish seven bacterial consortia. In general, researchers typically inoculate different species with an initial inoculation ratio of 1:1, which is based on either the optical density at 600 nm (OD_600_) or cell numbers; the effects of different inoculation ratio are rarely considered [32,33,34]. This study aims to explore the effects of different inoculation ratios on PAHs degradation. Therefore, the following ratios were selected in this study: H1 (ZL7:MI = 1:1), H2 (Q3:ZL7 = 1:1), H3 (Q3:MI = 1:1), H4 (Q3:ZL7: MI = 1:1:1), H5 (Q3:ZL7:MI = 2:2:1), H6 (Q3:ZL7:MI = 1:2:2) and H7 (Q3:ZL7:MI = 2:1:2).

PAHs degradation efficiency of the single strain and the bacterial consortia were evaluated in 150 mL flasks containing 50 mL of MSM added with 10% (*v/v*) inoculant. The MSM was enriched with either PYR at 50 mg/L as a single substrate or a mixture of PHE, PYR and B[a]P at 50 mg/L with B[b]F at 10 mg/L as a complex substrate. There were three replicates for each treatment. The flasks were incubated at 37 °C, on a rotating shaker at 180 rpm for 8 days. The treatment without PAHs degrading bacteria was used as abiotic controls. The degree of degradation of PAHs was determined after 8 days of incubation.

### 2.3. Immobilization of the Bacterial Consortium H6 Onto Biochar and SEM Observation

Biochar was prepared via the pyrolysis of rice straw at 500 °C for 4 h. The physico-chemical properties and 16 US EPA PAHs concentration of biochar are provided in Supplementary Materials (see Supplementary Materials Table S3 for quantification details). Bacterial consortium H6 was immobilized onto biochar, and then the immobilization efficiency was verified by Scanning Electron Microscope (SEM) according to the method described by Xiong et al. [11]. Briefly, the biochar (1 g) was passed through a 1 mm sieve and then placed in a 100 mL Erlenmeyer bottle with 10 mL LB medium. The Erlenmeyer bottle was sterilized at 121 °C for 30 min. The immobilization of bacterial consortium H6 suspensions were accomplished using an orbital shaker at 80 rpm 30 °C for 48 h. Separation of the mixtures (immobilized bacterial consortia with biochar and free bacterial solution) was done using 75 µm screen filtration [11]. Some free cells will be attached to the surface of biochar after immobilization, so in order to avoid interference, rinse the carrier material on the screen with 15 mL sterile water three times.

The composites were then fixed in 2.5% glutaraldehyde for 4 h at 4 °C. The samples were then rinsed twice with 0.1 M phosphate buffer pH (7.2–7.4) for 10 min. Dehydration was carried out in a graded ethanol/water series of 30, 50, 70, 90, and 100%, at 20 min for each concentration. The samples were then dried for 2.5 h using a point dryer (Samdri-PVT-3D). The dried composite material was observed by SEM. SEM images of samples were shown in Figure S1.

### 2.4. Bioremediation Experiment

Different bioremediation treatments were carried out in 250 mL conical flasks containing 10 g of soil from the coke plant (dry weight) (Table 1). A soil-water ratio of 1 g:10 mL was maintained by adding sterile water every 3 days to keep the weight of the system constant. The proportion of inoculated bacterial consortium H6 was 10% (*w*/*w*). The concentration of glucose and SDBS were 100 mg/L and 200 mg/L, respectively. Each flask was incubated at 37 °C on an orbital shaker at 180 rpm for 24 days. Soil suspension was then centrifuged at 6000 rpm for 20 min to achieve solid-liquid separation. There were three replicates per treatment.

### 2.5. Analysis of PAHs Concentrations

The determination of PAH concentrations in liquid medium was completed with reference to the Chinese standard (HJ 478-2009). PAHs were extracted using hexane and shaken at 180 rpm for 30 min. After static stratification, the organic and aqueous phases were separated. The above steps were repeated three times, and the extracts were concentrated using rotary evaporation. Finally, the samples were volumetrically adjusted to 1 mL with methanol (chromatographically pure) and filtered using the lead peracetate membrane (0.45 µm pore size). PAHs analysis was carried out by HPLC (Hitachi L-2000) at a fixed wavelength (254 nm) and a column temperature of 35 °C. A total of 20 µL of filtered solutions was injected into a C18 reverse chromatographic column (250 mm × 4.6 mm, 5 µm). A methanol/water (9:1/*v*:*v*) was used as the mobile phase at a flow rate of 1 mL/min, and the run time was 25 min. PAHs degradation efficiency (E%) was calculated as follows (1):

(1) $$E\%=\frac{C_{0}-C_{t}}{C_{0}}\times 100\%$$

where *C*_0_ is the residual PAHs (mg/L) in the abiotic control at the end of the assay, and *C_t_* is the residual PAHs (mg/L) in the treatment at the end of the assay.

The concentrations of PAHs in aqueous phases of the slurry system were determined according to the procedure reported by Ahn et al. [35]. The flocculate suspended solids in aqueous phases were removed by vibration and centrifugation after adding aluminum sulfate. The clear supernatant and hexane (15 mL) were added to 250 mL conical flasks, and then the flasks were shaken on a rotary shaker at 180 rpm for 30 min. The extracts were separated by separatory funnels. All extracts were collected after extraction, three times. Concentration, purification, and GC-MS determination of the extract were described in Supplementary Materials.

PAHs in soil phases of the slurry system were extracted by fast solvent extraction instrument Diane ASE 350 [36]. The extraction conditions were 100 °C, 1500 psi pressure, static extraction 5 min, cycle 3 times, solvent elution 70% (volume), nitrogen purge 100 s, and sample extraction was completed in 30 min. Concentration and purification of the extract and determination with GC-MS were described in Supplementary Materials.

### 2.6. Analysis of Soil Enzyme Activities

Soil dehydrogenase and polyphenol oxidase activity were quantified to assess the enzyme activity in different treatments [37,38]. The dehydrogenase activity was measured using 2,3,5-triphenyltetrazolium chloride (TTC) (as per the method reported by Lu et al.) [39]. The activity of dehydrogenase was expressed as triphenyl cresol (TPF) produced per kg of soil per hour (mg TPF kg^−1^ soil h^−1^). The determination of polyphenol oxidase activity was carried out as described by Perucci et al. [40]. Briefly, 1 g of soil sample, 3 mL reaction liquid (equivalent volume ratio mixture of 0.2 M catechol and 0.2 M proline) and 2 mL phosphate buffer (0.1 M, pH = 6.5) were added into the test tube and incubated at 30 °C for 10 min. After the reaction, all the samples were transferred into ice, and 5 mL ethanol was added to each reaction tube to terminate the reaction. The test tubes were centrifuged at 5000 rpm for 5 min. The absorbance of supernatant was measured at 525 nm. The activity of polyphenol oxidase was expressed as the amount of o-diphenol (mmol) oxidized within 10 min in 1 kg dry weight soil (dry weight).

### 2.7. Statistical Analysis

Data were analyzed using SPSS 13.0(IBM Corporation Software Group, Somers, NY, USA), and differences with a *p* value < 0.05 were considered statistically significant. Data were compared by one-way ANOVA (Duncan test) followed by either the LSD test or the Dunnett’s T3 test, depending on whether equal variances were or were not assumed, respectively, to compare differences between multiple groups.

## 3. Results and Discussion

### 3.1. PAH Degrading Strains and Antagonistic Effect

**Figure 1:**
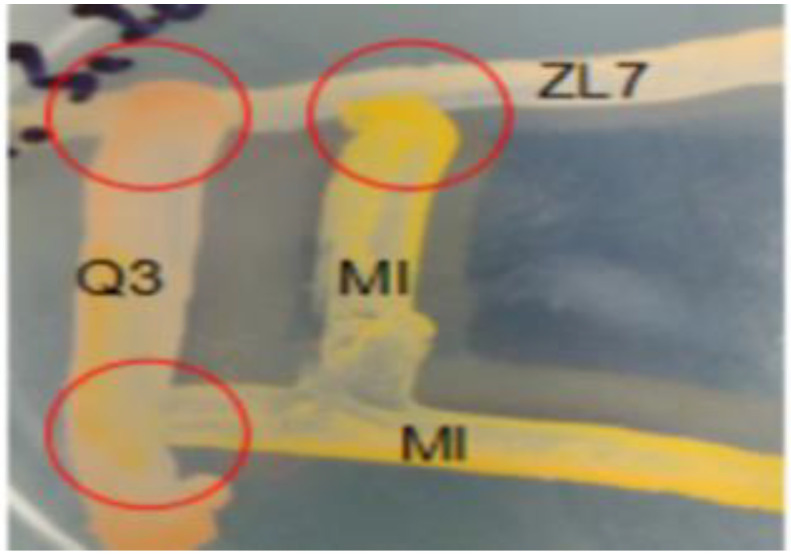
Antagonistic test of tested strains.

The antagonistic test showed no cross-inhibition among three strains (Figure 1), which indicated that the strains (Q3, MI and ZL7) had the potential for establishing the PAHs degrading bacterial consortia. It has been reported that the degradation efficiency of PAHs by inoculation of single bacteria was limited [12]. In contrast, bacterial consortia with multi-species can form a complex, adapted and stable micro-ecosystem by symbiotic proliferation and synergistic metabolism, which can degrade various organic pollutants [14,15,41]. The consortium construction was based on the following principles: (1) the composition of the artificial consortium was highly efficient degrading bacteria; (2) there is no antagonism between different strains; (3) the performance of PAH degradation is better than that of each single strain. In this study, mutual inhibition competition was not observed among Q3, MI and ZL7. The main degradable substrate of MI was LMW PAHs, while ZL7 and Q3 were able to utilize both LMW and HMW PAHs. The biochemical properties of the tested PAHs degrading strains are shown in Table S2. The results implied that the three strains were suitable for establishing the PAHs degrading bacterial consortia.

**Figure 2:**
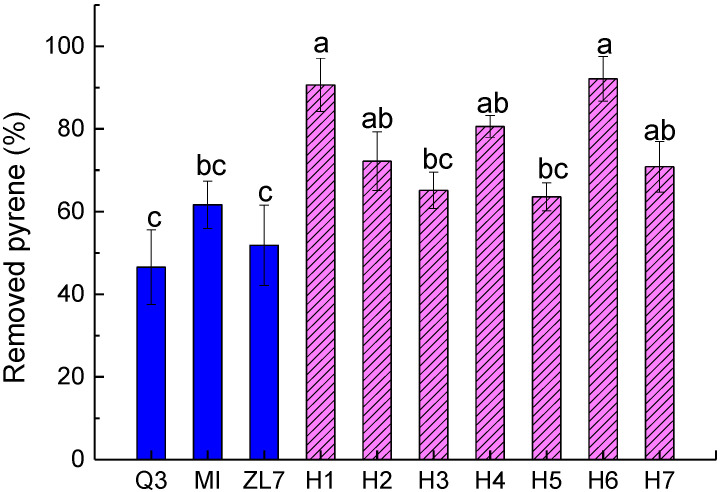
Pyrene degradation by single strain and seven bacterial consortia. Error bars represent the standard deviation (*n* = 3). Different letters indicate the mean difference is significant between treatments at 0.05 level. H1 (ZL7:MI = 1:1), H2 (Q3:ZL7 = 1:1), H3 (Q3:MI = 1:1), H4 (Q3:ZL7:MI = 1:1:1), H5 (Q3:ZL7:MI = 2:2:1), H6 (Q3:ZL7:MI = 1:2:2) and H7 (Q3:ZL7:MI = 2:1:2).

The bacterial consortia showed a higher PYR degradation ability than that of each single strain (Figure 2). The degradation efficiency of PYR by the PAH degrading bacterial consortia ranged between 64–92%, whereas the degradation efficiency of PYR by the single strains (Q3, ZL7 and MI) ranged between 47–59%. The results showed that the interaction in the bacterial consortia could facilitate the degradation of PAHs. Moreover, the degradation rate of PYR by the bacterial consortia H1 (ZL7:MI = 1:1) and H6 (Q3:ZL7:MI = 1:2:2) were significantly higher than the other strains and consortia (*p* < 0.05), reaching as high as 91% and 92%, respectively. Wanapaisan et al. also reported a significantly higher PYR degradation ratio of a mixed consortia than single strain, the cooperation among the strains should be considered in the degradation of PAHs [16]. Some strains can use the metabolites of other strains to improve their metabolic activity. Zafra et al. constructed bacterial consortia which could degrade not only PYR but also PHE and BaP [17]. Thus, it is necessary to further investigate the degradation potential of single strain or consortium on other PAHs.

### 3.2. Degradation Efficiency of PAHs Mixtures by single Strain and Bacterial Consortia in Liquid Medium

**Figure 3:**
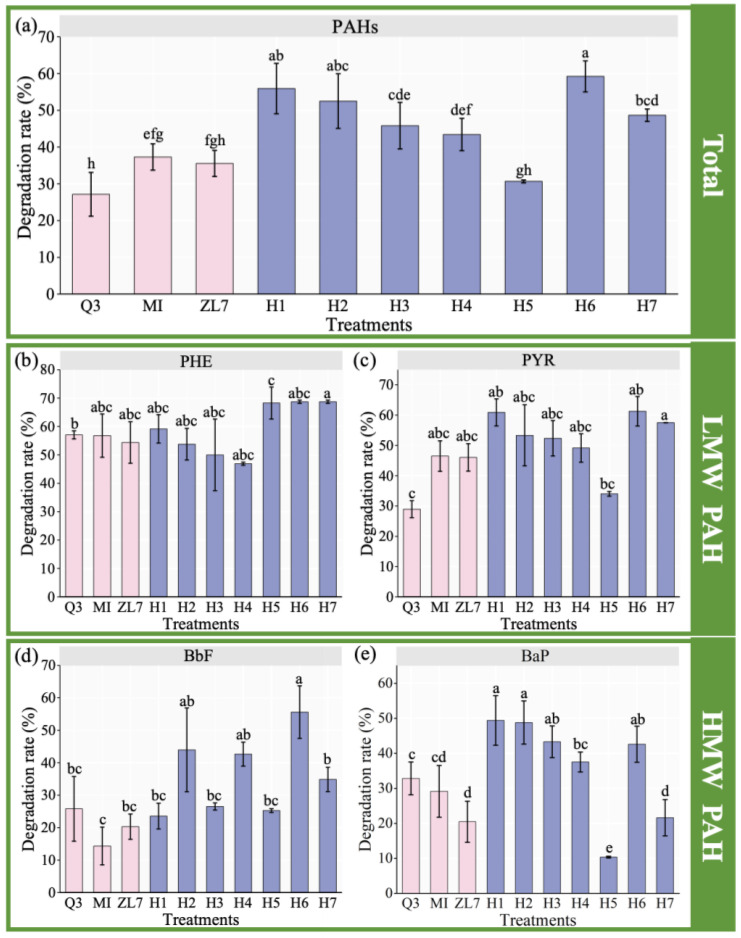
Degradation rate of the PAH mixture by single strains (ZL7, MI and Q3) and the microbial consortia in liquid medium. (**a**) Degradation rate of the total PAH mixture; (**b**) Degradation rate of phenanthrene(PHE) in PAHs mixture; (**c**) Degradation rate of pyrene(PYR) in PAHs mixture; (**d**) Degradation rate of benzo[b]fluoranthene(BbF) in PAHs mixture; (**e**) Degradation rate of benzo[a]pyrene(BaP) in PAHs mixture; Error bars represent the standard deviation (*n* = 3). Different letters indicate the mean difference is significant between treatments at 0.05 level. H1 (ZL7:MI = 1:1), H2 (Q3:ZL7 = 1:1), H3 (Q3:MI = 1:1), H4 (Q3:ZL7:MI = 1:1:1), H5 (Q3:ZL7:MI = 2:2:1), H6 (Q3:ZL7:MI = 1:2:2) and H7 (Q3:ZL7:MI = 2:1:2).

To explore the microbial degrading performance of mixed PAHs, the degradation efficiency of the PAH mixture solution (50 mg/L PHE, 50 mg/L PYR, 50 mg/L BaP, 10 mg/L BbF) by the single strains and the established consortia was studied. Total PAHs biodegradation of Q3, MI and ZL7 in liquid medium were 27%, 37% and 36%, respectively, in 8 days (Figure 3a). The degradation rate of PAHs by MI was significantly higher than Q3 (*p* < 0.05). The degradation rate of PHE in mixed PAHs contaminated solution by Q3, MI and ZL7 was 57%, 57% and 54%, respectively (Figure 3b). The degradation rate of PYR in mixed PAHs simulation solution by Q3, MI and ZL7 was 29%, 46% and 46%, respectively (Figure 3c). The degradation rate of BbF in the PAHs mixture by Q3, MI and ZL7 was 26%, 14% and 20%, respectively (Figure 3d). The degradation rate of BaP by Q3, MI and ZL7 was 33%, 29% and 20%, respectively (Figure 3e). However, MI and ZL7 could not degrade BaP when it was present as the sole carbon source (Table S2). Interestingly, the presence of PHE, PYR and BbF promoted the degradation of BaP. Gong et al. reported similar results that the addition of PHE promoted the degradation of PYR, which was due to the oxidative degradation of PHE leading to the activation and enhancement of enzymes with potential degradation properties to PYR, thus promoting the degradation of PYR [42].

All bacterial consortia except for H5 showed a higher degradation capability of total PAHs than each single strain. Among the seven bacterial consortia, the highest degradation rate of total PAHs was 59% by H6, which was 22–32% higher than that by single strain (Figure 3a). The highest degradation rate of single aromatic compound was 69% for PHE by H7 (12–15% higher than single strain), 49% for BaP by H1 (16–29% higher than single strain), 61% for PYR (15–32% higher than single strain) and 56% for BbF (30–42% higher than single strain) by H6 (Figure 3). These results indicated the addition of strain Q3 improved the degradation of other kinds of PAHs and total PAHs by bacterial consortia H6. The bacterial consortium H6 (Q3:ZL7:MI = 1:2:2) was the most effective consortium in degrading PAHs in this study. The previous study found that the initial inoculation ratio influences the structure, function and even the interaction of bacterial consortium, which may contribute to their degradation ability. The other environmental factors may possibly influence the performance of different consortia in this study, but further experiments should be carried out to explore the detailed mechanism in the future study [43]. The possible reason is the synergetic metabolism among different PAH degrading bacteria. The metabolites of strain A could be metabolized directly by strain B, which could reduce the toxic risk of intermediate products to the degrading bacteria and improve the degradation potential of PAHs by bacterial consortia [44]. Furthermore, the metabolites of PAHs by strain A could be used as nutrients by strain B, which could promote the growth of strain B and reduce the inhibition of intermediate metabolites accumulation on the degradation efficiency [45]. Meanwhile, the stable bacterial consortia formed by the strains with the co-metabolism relationship may drive out other strains unrelated to the degradation process in the environment, thus increasing the abundance of degrading related strains and improving the metabolic efficiency [46]. Based on the above results, there were no consortium with a significant higher degradation efficiency for PAHs than H6. Therefore, bacterial consortium H6 had the optimal performance among different treatments.

These results were consistent with previous studies that PAH biodegradation efficiency by constructed bacterial consortia was higher than that by single strain inoculant. Similar results were reported by Kumaria et al. [47], who constructed a bacterial consortium with *Stenotrophomonas maltophilia*, *Ochrobactrum anthropi*, *Pseudomonas mendocina*, *Microbacterium esteraromaticum* and *Pseudomonas aeruginosa*, and showed that enhanced biodegradation rate towards naphthalene 89% (10 mg/L), fluorene 64% (1.9 mg/L), phenanthrene 81% (3.5 mg/L) and benzo[b]fluoranthene 73% (6.5 mg/L) in liquid medium in 45 days, which were 7%, 16%, 14% and 12% higher than single strain, respectively. Compared with the previous studies, the bacterial consortium H6 constructed in this study could tolerate higher concentration of PAHs and showed a higher degradation rate for mixed PAHs. The degree of improvement of the PAH degradation by bacterial consortia was higher than that by single strain in this study and even the above studies. Isaac et al. also constructed sixteen bacterial consortia with 5 strains, and C15 mixed culture removed 100% naphthalene (12.8 mg/L) and phenanthrene (17.8 mg/L), and showed the highest degradation (close to 42%) rate of pyrene (20.2 mg/L) after 7 days of incubation in liquid medium with mixed PAHs. However, in this study, the degradation rate of PYR (61%) was much higher after 8 days of incubation [48]. This result suggested that bacterial consortium H6 was effective in the remediation of PAHs contaminated soil. So, bacterial consortium H6 was selected for subsequent soil experiments for its higher degradation rate of PAHs.

### 3.3. Bioremediation of PAHs in Contaminated Soil

**Figure 4:**
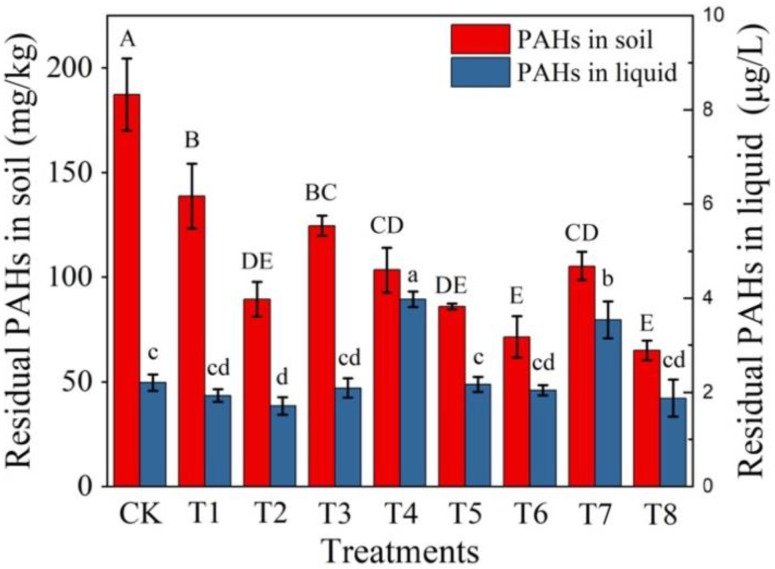
PAHs in soil and liquid phase after incubation for 24 days. Different letters (A–E and a–d) indicate significant differences in the concentrations of PAHs among different treatments according to the one-way analysis of variance (ANOVA) test followed by Tukey’s post hoc tests.

**Figure 5:**
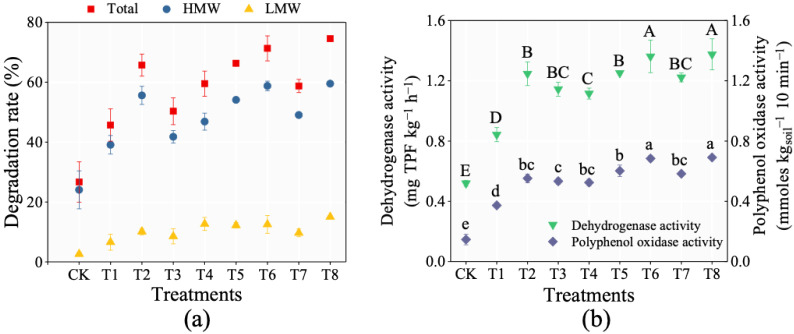
(**a**) Degradation rates of total, HMW, and LMW PAHs. (**b**) Soil polyphenol oxidase and dehydrogenase activities after incubation for 24 days. Different letters (A–E and a–d) indicate significant differences in the concentrations of PAHs among different treatments according to the one-way analysis of variance (ANOVA) test followed by Tukey’s post hoc tests.

To further explore the degradation efficiency of H6 in the contaminated soil, H6 was inoculated into soil for 24 days of incubation. The residual PAHs in T1 is 138.7 mg/kg after 24 days of incubation, which were significantly lower than that in CK (187.3 mg/kg) (Figure 4, Table S4). In addition, there were different degradation efficiencies for PAHs with different molecular weights in T1 (i.e., 39% for HMW PAHs and 7% for LMW PAHs, respectively). Soil dehydrogenase and polyphenol oxidase activities were used to examine the degradation potential of bacterial strains and consortia constructed towards PAHs. Soil polyphenol oxidase and dehydrogenase activities in T1 were significantly higher than those in CK (Figure 5b). Soil dehydrogenase activity is usually related to the presence of viable microorganisms and their oxidative capabilities, which is used to represent soil microbial activity [49]. Soil polyphenol oxidase activity has been linked with heterotrophic aromatic hydrocarbon degrading microbial populations, which is usually used to indicate the activity of PAH-degrading bacteria in soil [38]. All of the above results showed that the addition of bacterial consortium H6 significantly enhanced the degradation rate of PAHs in soil.

Different strengthening remediation strategies were used to further improve the degradation efficiency of PAHs in soil by H6. As for soil phase, the residual PAHs in T2 and T4–T8 were significantly lower than that in T1. As for liquid phase, the residual PAH in T4 and T7 were significantly higher than that in T1 after 24 days of incubation (Figure 4). Compared with T1, other treatments (except CK) had no significant effects on the degradation of LWH PAHs but had significant effects on HWH PAHs (Figure 5a). In particular, the degradation rates of HMW PAHs in the treatments with immobilization (i.e., T2, T5, T6 and T8) were significantly higher than those in other treatments. T8 (glucose + SDBS + immobilization + bacterial consortium H6) showed the highest degradation rate of PAHs reaching 75% after 24 days of incubation, and the degradation rate of HWM PAHs reached 60%. Meanwhile, the activities of dehydrogenase and polyphenol oxidase in T8 were higher than those in other treatments (Figure 5b). These results are similar to Sun et al., who showed the degradation rate towards total PAHs reached 44%, and that of HWM PAHs (4–6 rings) reached 55% after 175 days of incubation, added together with PAHs degradation bacteria and carbon source [50]. In this study, although the total PAHs content in the tested soil is less than the above study, the content of HMW PAHs, the degradation rates of total PAHs and HMW PAHs were higher than those in the above study.

The above results may involve multiple possible mechanisms, with different strengthening remediation strategies combined to achieve optimal performance. Glucose promoted the growth of functional microorganisms and stimulated the metabolism of PAHs by indigenous microorganisms, thus promoting the degradation of PAHs [51]. In addition, glucose might promote microorganism to absorb more PAHs, thus reducing the distance between PAHs and degradation bacteria [52]. Yang et al. found similar results that 16% of PHE and 15% of PYR were adsorbed on microorganism biomass in the presence of glucose, then further promoted PAHs degradation [53]. Surfactants SDBS might be used as carbon sources by indigenous microorganisms, increasing the number of PAHs degradation bacteria or directly stimulating the activity of degradation bacteria. Wang et al. found similar results that SDBS significantly increased microbial biomass and removed PAHs [13]. Meanwhile, SDBS might increase the bioavailability of PAHs by enhancing the mobility of pollutants or shortening the distance between pollutants and microbial cells [54]. In this study, the residual PAHs in liquid phase of test groups with SDBS addition and no immobilization (T4 and T7) were significantly higher than those in other test groups and control (Figure 4), which confirmed the above mechanisms. Immobilization could protect the strains from higher concentrations of PAHs or surfactant, thereby reducing the toxicity from the pollutant or surfactant [55]. In addition, biochar exhibited stronger affinity with organic pollutants than soil organic matter, thus, biochar mightadsorb PAHs in contaminated soil [56]. Therefore, microbes can be fully exposed to contaminants on the biochar. There were no significant differences between the control and treatment groups adding SDBS and immobilized bacterial consortia in liquid phase (Figure 4), which further confirmed the above assumptions.

## 4. Conclusions

In this study, a comprehensive technology combining microbial degradation consortia with strengthening remediation strategies was proposed for remediating soil with PAHs contamination. The degradation efficiency of PAHs by bacterial consortia was better than that of three single strains (MI, ZL7 and Q3), and bacterial consortium H6 was the most effective consortium in degrading the PAH mixture in liquid phase. Furthermore, bacterial consortium H6 also showed better degradation effect of PAHs in aged soil. Immobilization is the most effective strategy for improving the remediation performance of PAHs in comparison with other strengthening strategies (e.g., addition of surfactants or nutrients). Nevertheless, immobilization combined with other strengthening measures can further improve the degradation of PAH. The highest degradation rate was observed in the treatment using immobilized microbial consortium H6 amended with glucose and SDBS, reaching 75%. The combination of bioremediation strategies, including simultaneous addition of SDBS and glucose as well as immobilization, is a promising way forward to enhance the degradation extent of aged PAHs in contaminated soils.

## Figures and Tables

**Table 1:** Experimental design of bioremediation.

Treatment Groups	Experimental Design
CK	Coke plant (CP) soil
T1	Bacterial consortium H6
T2	Immobilized bacterial consortium H6 on biochar
T3	Glucose + bacterial consortium H6
T4	SDBS + bacterial consortium H6
T5	Glucose + immobilized bacterial consortium H6 on biochar
T6	SDBS + immobilized bacterial consortium H6 on biochar
T7	SDBS + glucose + bacterial consortium H6
T8	SDBS + glucose + immobilized bacterial consortium H6 on biochar

## Data Availability

Some or all data and models that support the findings of this study are available from the corresponding author upon reasonable request.

## Supplementary Materials

The following supporting information can be downloaded at: https://www.mdpi.com/article/10.3390/ijerph20031766/s1, Table S1: The total concentrations of 16 US EPA PAHs in CP soil; Table S2: Biochemical properties of bacterial strains isolated from PAH contaminated soil; Table S3: Total concentrations of 16 US EPA PAHs in test biochar; Table S4: Residual PAH in CP soil with different treatments for 24 days; Figure S1: SEM images of biochar immobilized microbial composites. Reference [57] is cited in the supplementary materials.
