# Supplementary material for: Enhanced Bioremediation of Aged Polycyclic Aromatic Hydrocarbons in Soil Using Immobilized Microbial Consortia Combined with Strengthening Remediation Strategies
Source: Int J Environ Res Public Health. 2023 Jan 18;20(3):1766. doi: 10.3390/ijerph20031766 (PMC9914441; doi:10.3390/ijerph20031766)
Supplement: Supplementary file 1 [file ijerph-20-01766-s001.zip › ijerph-2105676-supplementary.pdf]

Supporting Information

Enhanced bioremediation of aged polycyclic aromatic hydrocarbons in soil using immobilized microbial consortia combined with strengthening remediation strategies

Haixuan Zhou[1†], Xiurong Gao[1,2†], Suhang Wang[1,2], Youchi Zhang[1], Frédéric Coulon[3], Chao Cai[1*]

[1] Key Lab of Urban Environment and Health, Institute of Urban Environment, Chinese Academy of Sciences, Xiamen 361021, China

[2] University of Chinese Academy of Sciences, Beijing 100049, China

[3] School of Water, Energy and Environment, Cranfield University, Cranfield MK43 0AL, UK

[†] These authors contributed equally to this work.

[*] Correspondence should be addressed to: ccai@iue.ac.cn (Chao Cai)

Supporting Methodology

Chemicals: Phenanthrene (PHE), pyrene (PYR), benzo (b) fluoranthene (BbF) and (BaP) benzo[a]pyrene (>97% purity), the surrogate standard mix contained naphthalene-d^{10}, acenaphthene-d^{10}, phenanthrene-d^{10}, chrysene-d^{12} and perylene-d^{12}, a standard solution of 16 US EPA PAHs, were purchased from AccuStandard (CT, USA). N-hexane, methanol, acetone, and dichloromethane (all HPLC-spectrograde) were purchased from Tedia (TC, USA). Internal standard hexamethylbenzene (99% purity) was acquired from Dr. Ehrenstorfer (Augsburg, Germany). All the inorganic reagents were of analytical grade (Sinopharm Chemical Reagent Co., Ltd, Shanghai, China).

Medium: Minimal salt medium (MSM) contained (g/L) KH_2PO_4 3.4 g, Na_2HPO_4 19.6 g, $(NH4)_2SO_4$ 1 g, $MgSO_4$ 97.4 mg and trace element, such as $CuSO_4$ 1 g, $ZnSO_4$ 1 g, $FeSO_4$ 1 g, $CaCl_2$ 1 g, H_3BO_4 1 g.

LB medium contained (g/L) Tryptone 10g, Yeast extract 5g, NaCl 10g.

LB solid medium was made by adding 20% agar.

The determination of physical and chemical properties of soil were according to the methods described in Lu (1999), and the specific procedures are as follows.

pH was measured in a suspension of soil/biochar with soil or biochar /water ratio of 1:2.5 (W/V). The content of soil organic matter was determined by potassium dichromate oxidation-volumetric method.

The contents of C, N, S, H and O elements in the soil/biochar were determined by Vario

Max elemental analyzer (VarioMax CNS, Germany).

The specific surface area and pore size of biochar were measured using 99.99% N_2 as adsorbent and at 1.036 bar saturated vapor pressure by automatic specific surface area, micropore and chemical adsorption instrument (ASAP 2020M+C).

PAH analysis in CP soil: A surrogate standard mix containing naphthalene-d^{10}, acenaphthene-d^{10}, phenanthrene-d^{10}, chrysene-d^{12} and perylene-d^{12} (25 μL) at concentration of 400 μg/mL was added to the soil phase prior to extraction. Following the US EPA Method 3630C, the extracts were concentrated to 1 mL by rotary vacuum evaporation at 36°C. Then 15 mL of hexane were added and further concentrated to 1 mL. An internal standard hexamethylbenzene 15 μL (10 μg/mL in acetone) was added to the final solution added before analysis by GC-MS.

Table S1 The total concentrations of 16 US EPA PAHs in CP soil

PAHs	Abbreviation	Ring number	Concentration(mg/kg)
Naphthalene	NAP	2	1.89 ± 0.27
Acenaphthylene	ANY	2	0.44 ± 0.15
Acenaphthene	ANA	3	2.74 ± 0.37
Fluorene	FLU	3	1.58 ± 0.29
Phenanthrene	PHE	3	8.99 ± 1.73
Anthracene	ANT	3	1.88 ± 0.26
Fluoranthene	FLT	4	21.91 ± 3.87
Pyrene	PYR	4	22.46 ± 2.61
Benzo(a)anthracene	BaA	4	20.78 ± 2.67
Chrysene	CHR	4	23.52 ± 3.35
Benzo(b)fluorene	BbF	5	31.87 ± 4.43
Benzo (k)fluorene	BkF	5	14.91 ± 2.05
Benzo(a)pyrene	BaP	5	32.05 ± 3.67
Indeno(1,2,3-c,d)pyrene	ICP	6	7.36 ± 0.76
Dibenzo(a,h)anthracen	DBA	6	36.03 ± 3.11
Benzo(g,h,i)perylene	BPE	6	27.07 ± 2.03
Total PAHs			255.47 ± 22.46

The physico-chemical properties of the biochar were as follows: pH 10.0, total N 1.47 %, total C 47.6 %, total O 16.1 %, total H 2.25 %, specific surface area 2.98 m^2/g.

The physico-chemical properties of soil were as follows: pH 8.6, total N 0.24 %, total C 16.6 %, total S 0.56 %, total Organic matter 8.20 %.

Table S2 Biochemical properties of bacterial strains isolated from PAH contaminated soil

Item	ZL7	MI	Q3
Colony color	Pink	Light-yellow	Orange
Colony surface	Dry smooth	Dry smooth	Dry smooth
Shape of cells	Short rod	Short rod	Globular
Gram staining[a]	+	+	+
Spore staining[a]	-	-	-
Degradable PAHs[b]	PHE, PYR, BaA, BbF, CHR and BkF	NAP, FLU, PHE, ANT, FLT, BbF and PYR	PHE, PYR, BaP, BbF, BaA, BkF, DBA, BPE and CHR

[a] +, Positive; -, Negative.

[b] Naphthalene (NAP), fluorene (FLU), phenanthrene (PHE), anthracene (ANT), fluoranthene (FLT), pyrene (PYR), benzo[a]pyrene (BaP), benzo[a]anthraxcene (BaA), benzo[b]fluoranthene (BbF), benzo[k]fluoranthene (BkF), dibenzo[a,h]anthracene (DBA), benzo[g,h,i]perylene (BPE) and chrysene (CHR).

Table S3 Total concentrations of 16 US EPA PAHs in test biochar

PAHs	Abbreviation	Ring number	Concentration（mg/kg）
Naphthalene	NAP	2	0.08 ± 0.003
Acenaphthylene	ANY	2	0.15 ± 0.007
Acenaphthene	ANA	3	0.01 ± 0.0001
Fluorene	FLU	3	0.05 ± 0.0002
Phenanthrene	PHE	3	0.09 ± 0
Anthracene	ANT	3	0.17 ± 0.02
Fluoranthene	FLT	4	0.14 ± 0
Pyrene	PYR	4	0.15 ± 0.007
Benzo(a)anthracene	BaA	4	0.28 ± 0.009
Chrysene	CHR	4	0.34 ± 0.01
Benzo(b)fluorene	BbF	5	0.24 ± 0.007
Benzo (k)fluorene	BkF	5	0.37 ± 0.02
Benzo(a)pyrene	BaP	5	0.31 ± 0.002
Indeno(1,2,3-c,d)pyrene	ICP	6	0.47 ± 0.02
Dibenzo(a,h)anthracen	DBA	6	0.53 ± 0.003
Benzo(g,h,i)perylene	BPE	6	0.35 ± 0.01
Total PAHs			3.72 ± 0.04

Table S4 Residual PAH in CP soil with different treatments for 24 days

PAHs	residual PAH (mg/kg)												
	CK	T1	T2	T3	T4	T5	T6	T7	T8	T9	T10	T11	T12
NAP	1.09±0.35	1.42±0.21	1.48±0.25	0.73±0.16	1.32±0.29	0.47±0.16	0.72±0.25	1.30±0.45	1.86±0.64	1.04±0.02	1.06±0.95	0.79±0.29	1.05±0.49
ANY	0.46±0.12	0.36±0.06	0.46±0.08	0.34±0.07	0.36±0.06	0.28±0.07	0.43±0.05	0.34±0.03	0.65±0.16	0.41±0.14	0.35±0.30	0.29±0.29	0.23±0.05
ANA	2.60±0.54	2.47±0.68	2.49±0.33	1.79±0.36	2.19±0.52	1.26±0.52	2.31±0.38	1.87±0.15	3.16±1.43	2.29±0.32	1.90±0.93	1.50±0.17	1.33±0.20
FLU	1.91±0.34	1.74±0.52	1.84±0.31	1.63±0.15	1.68±0.35	1.15±0.28	1.70±0.12	1.38±0.10	2.56±0.32	1.72±0.23	1.32±0.98	1.12±0.11	0.99±0.18
PHE	8.08±0.93	6.83±1.48	5.66±0.92	6.92±1.08	4.91±0.76	4.43±1.15	6.29±1.40	5.27±0.62	5.64±0.39	4.87±1.31	3.70±0.59	4.55±0.50	3.27±0.31
ANT	1.85±0.30	1.44±0.43	1.11±0.17	1.48±0.28	1.10±0.18	0.96±0.27	1.28±0.16	1.14±0.10	1.07±0.15	1.13±0.25	0.74±0.11	0.91±0.07	0.65±0.10
FLT	18.02±2.26	12.71±1.86	14.76±1.86	13.02±1.82	13.27±0.64	9.86±1.81	12.08±1.92	10.72±0.93	23.10±2.47	13.74±2.49	11.17±2.49	9.00±0.67	7.45±1.62
PYR	21.19±1.49	18.08±1.83	13.15±1.45	14.99±1.76	11.96±0.09	11.08±1.79	15.25±2.66	15.10±2.25	13.60±2.10	10.83±3.07	9.62±1.90	12.49±1.29	8.50±1.37
BaA	14.42±2.11	11.47±0.99	9.17±0.60	10.89±1.70	8.28±0.47	8.28±0.88	9.38±1.41	8.80±0.72	10.14±0.99	6.57±0.72	5.73±0.88	7.45±0.58	4.58±0.59
CHR	17.36±2.00	13.42±1.89	10.68±0.91	12.56±1.95	9.48±0.16	9.96±0.88	11.83±1.40	10.93±0.68	12.17±1.59	6.99±2.91	7.26±0.82	9.06±0.63	3.93±2.13
BbF	19.56±2.92	15.26±1.80	12.98±0.47	14.44±2.25	11.95±0.84	11.44±1.12	13.18±1.12	11.43±0.08	13.18±0.75	8.91±1.02	7.53±1.82	9.27±0.68	6.62±0.80
BkF	7.23±0.92	5.48±0.30	4.76±0.06	5.27±0.64	4.27±0.38	4.20±0.43	4.87±0.67	5.26±2.22	4.89±0.26	3.99±1.25	4.40±1.26	5.30±1.43	3.68±1.02
BaP	22.57±3.43	17.16±1.43	13.69±0.80	13.75±1.66	13.74±1.57	11.58±0.61	13.82±0.78	11.00±1.68	12.25±0.37	8.37±1.31	7.76±0.84	9.36±0.60	7.41±0.73
ICP	23.36±3.72	12.50±2.80	14.49±0.65	12.12±3.14	16.28±2.15	13.15±2.35	15.57±0.71	6.36±1.68	10.86±1.32	5.79±2.34	4.87±1.39	6.56±1.65	6.99±1.07
DBA	25.35±2.05	16.94±1.58	16.33±0.49	16.02±2.55	16.30±1.24	13.85±2.35	16.55±0.30	13.34±0.47	12.64±1.06	10.07±1.08	5.03±1.72	7.59±1.53	7.55±0.68
BPE	2.24±0.33	1.41±1.58	1.58±0.38	1.68±0.52	1.64±0.15	1.45±0.43	1.67±0.28	1.16±0.25	1.30±0.08	0.85±0.06	0.87±0.32	0.79±0.03	0.78±0.28
Total PAHs	187.29±7.14	138.70±15.48	124.61±4.73	127.60±18.17	118.74±4.64	103.40±10.74	126.93±11.47	105.39±6.80	129.13±7.33	87.59±9.42	73.33±13.03	86.01±1.36	65.01±4.71

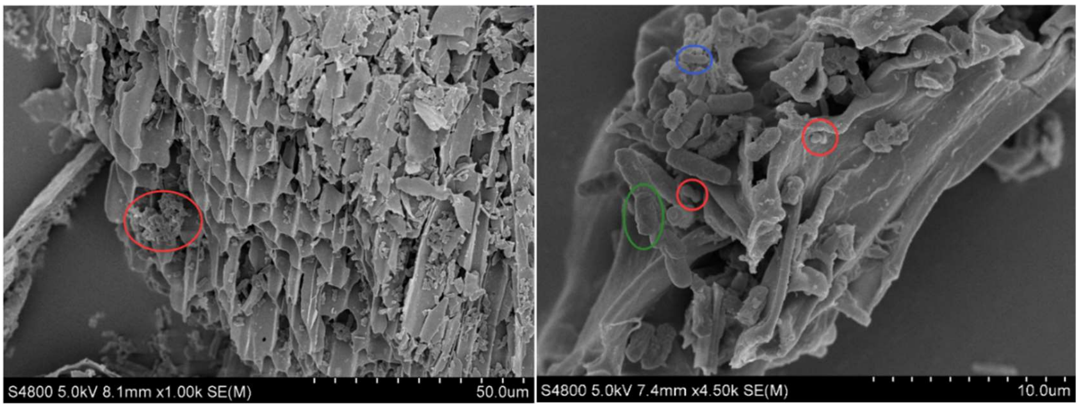

Figure S1 SEM images of biochar immobilized microbial composites

References

Lu, R.K. Soil argrochemistry analysis protocals. Beijing: China Agriculture Science Press: 1999.
